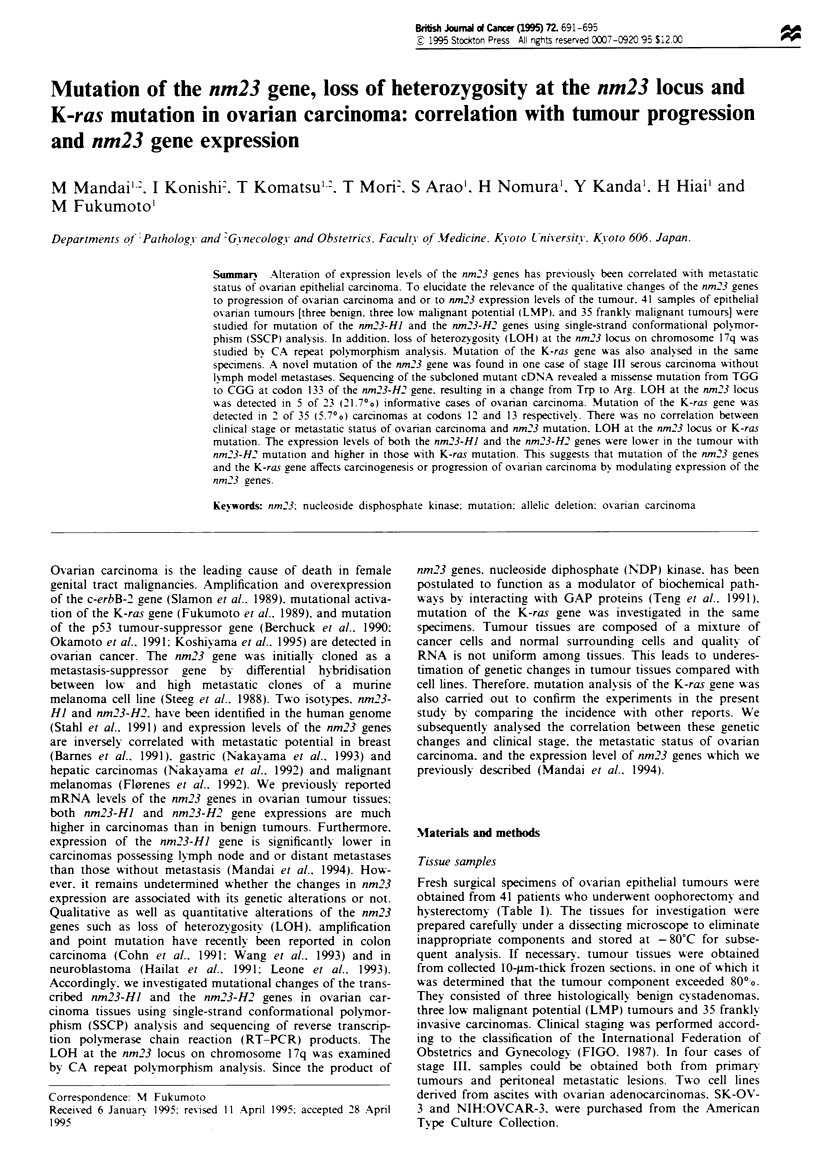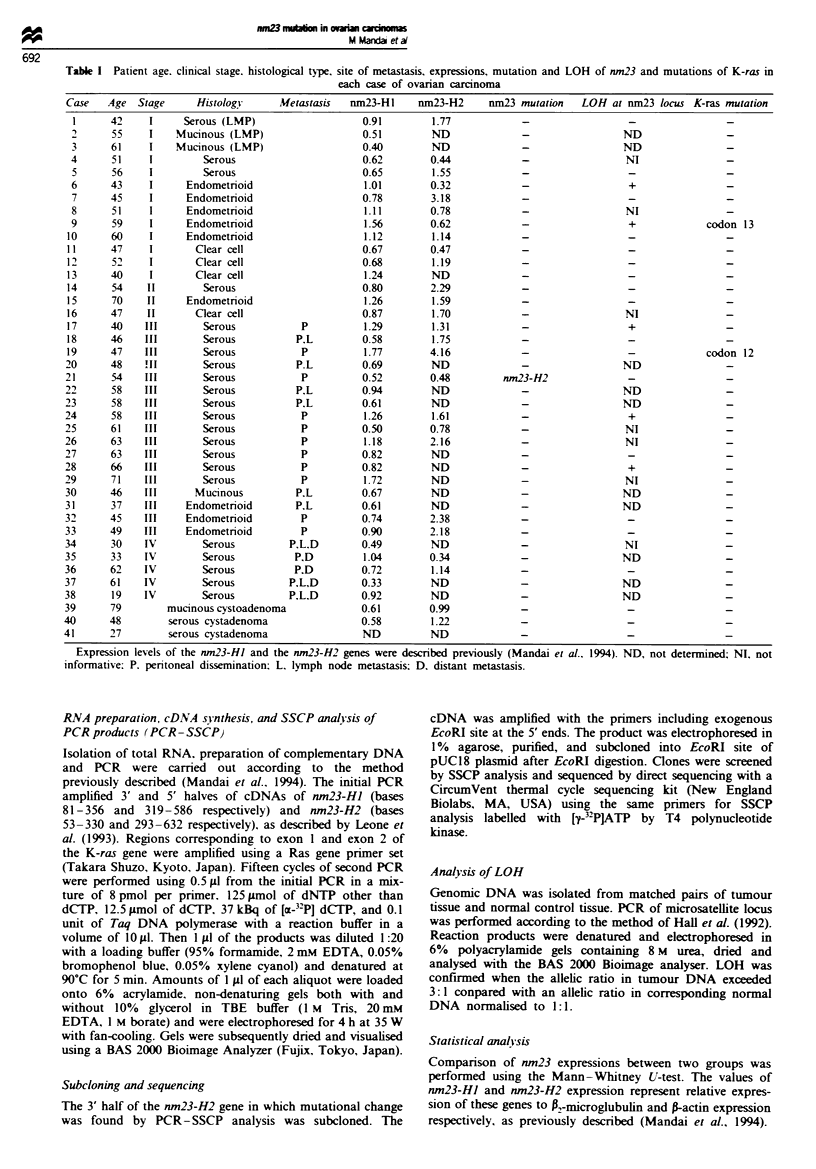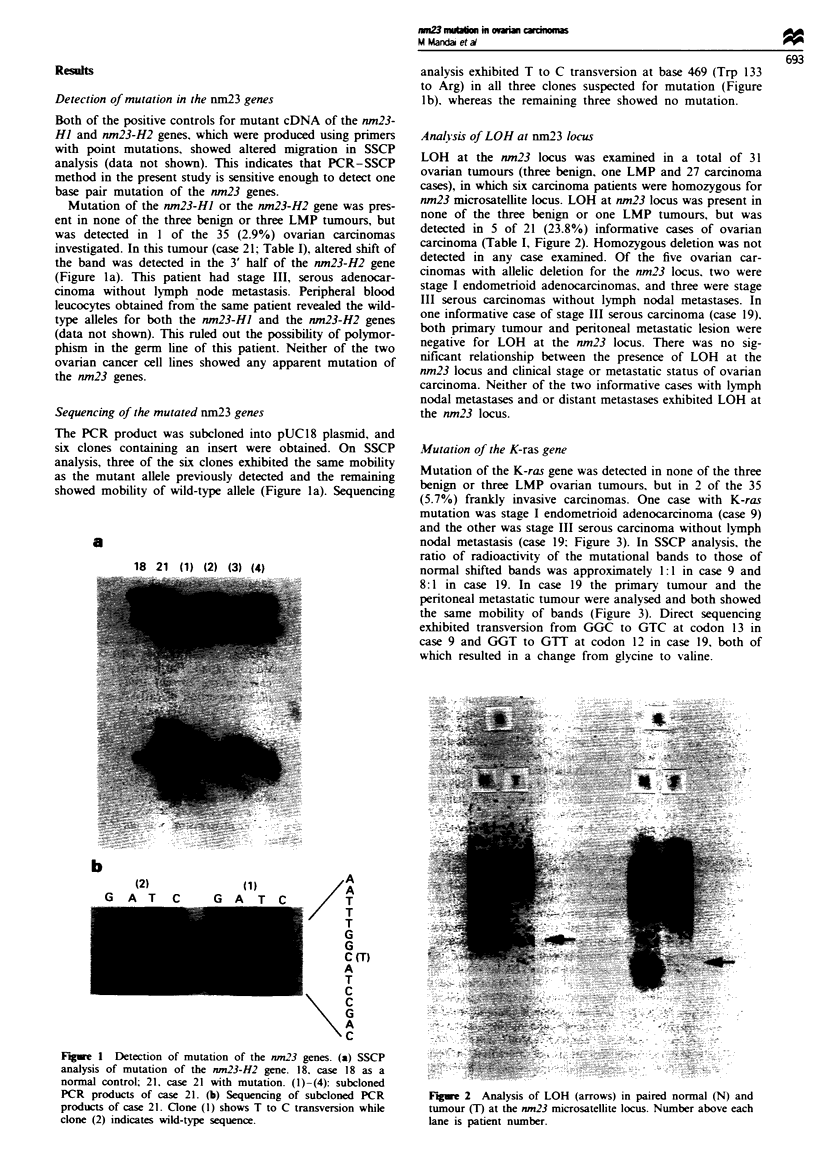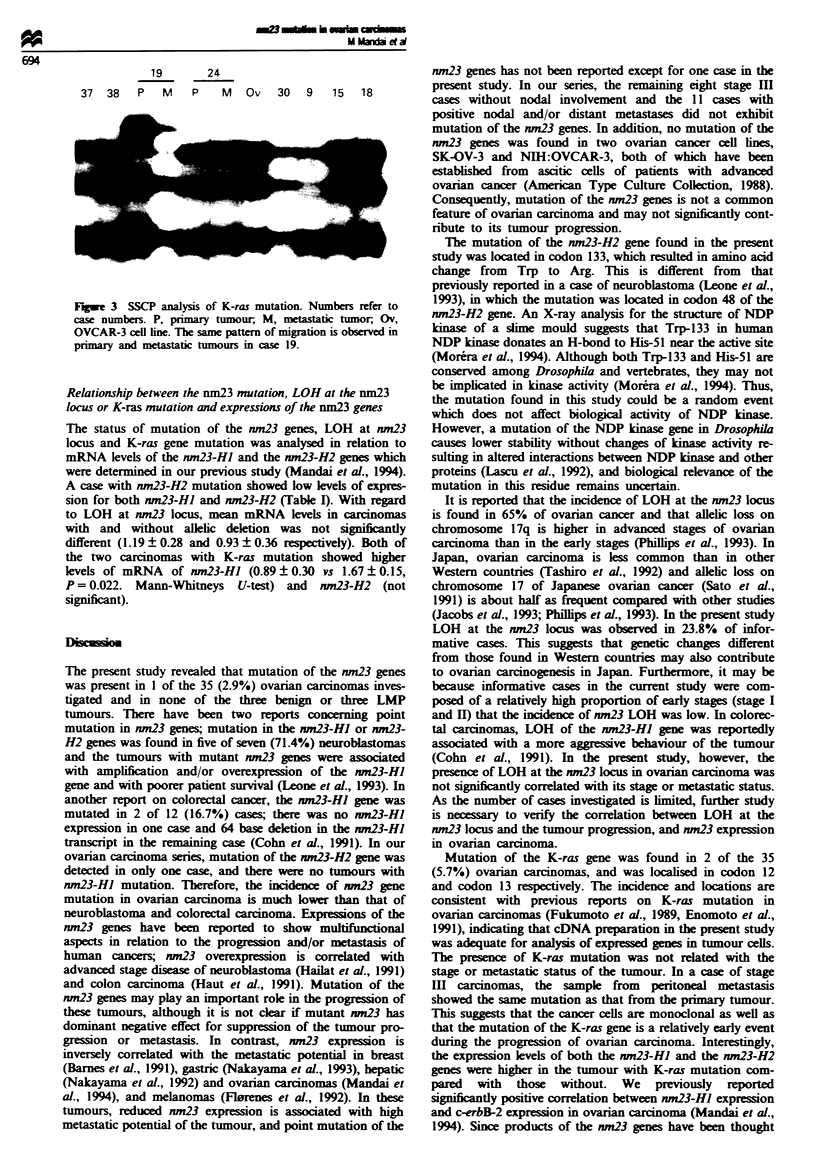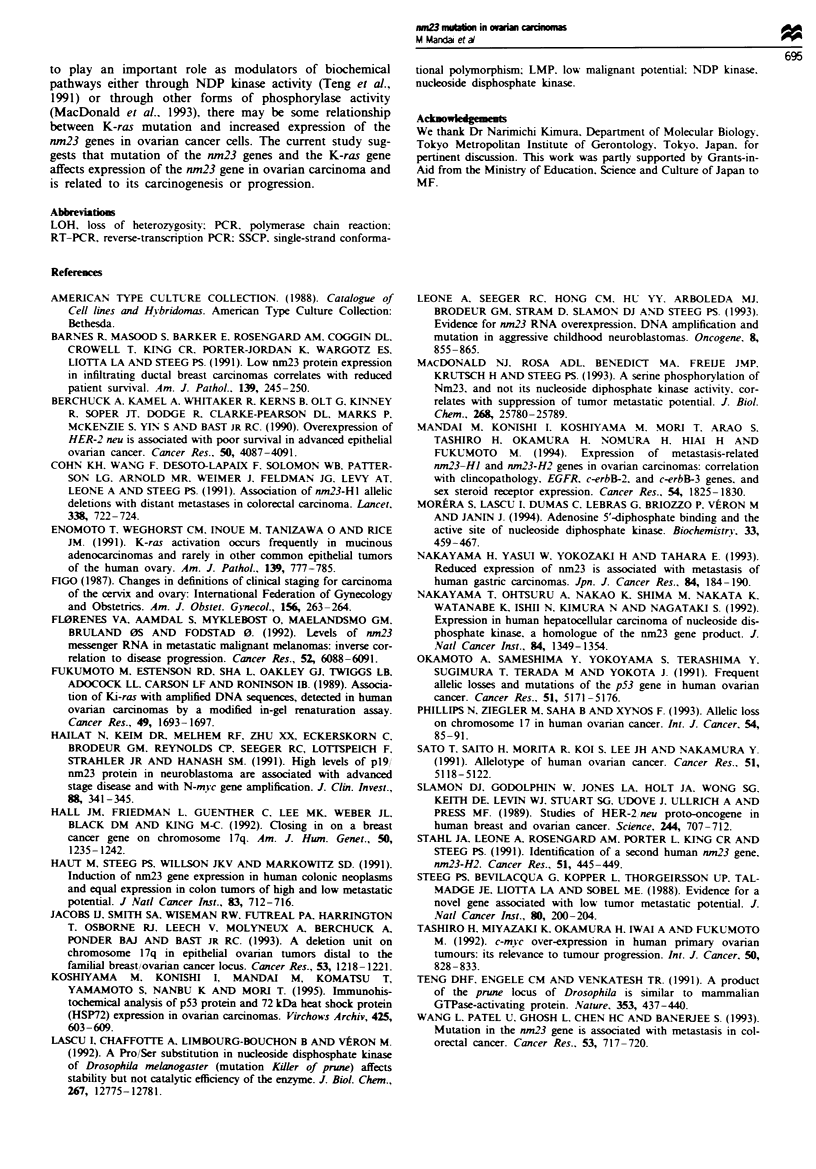# Mutation of the nm23 gene, loss of heterozygosity at the nm23 locus and K-ras mutation in ovarian carcinoma: correlation with tumour progression and nm23 gene expression.

**DOI:** 10.1038/bjc.1995.395

**Published:** 1995-09

**Authors:** M. Mandai, I. Konishi, T. Komatsu, T. Mori, S. Arao, H. Nomura, Y. Kanda, H. Hiai, M. Fukumoto

**Affiliations:** Department of Pathology, Faculty of Medicine, Kyoto University, Japan.

## Abstract

**Images:**


British Journal of Cancer (1995) 72, 691-695

© 1995 Stockton Press All rights reserved 0007-0920/95 $12.00

### Mutation of the *nm23* gene, loss of heterozygosity at the *nm23* locus and K-*ras* mutation in ovarian carcinoma: correlation with tumour progression and *nm23* gene expression

M Mandai[1,2], I Konishi[2], T Komatsu[1,2], T Mori[2], S Arao[1], H Nomura[1], Y Kanda[1], H Hiai[1] and M Fukumoto[1]

*Departments of [1]Pathology and [2]Gynecology and Obstetrics, Faculty of Medicine, Kyoto University, Kyoto 606, Japan.*

**Summary** Alteration of expression levels of the *nm23* genes has previously been correlated with metastatic status of ovarian epithelial carcinoma. To elucidate the relevance of the qualitative changes of the *nm23* genes to progression of ovarian carcinoma and or to *nm23* expression levels of the tumour, 41 samples of epithelial ovarian tumours [three benign, three low malignant potential (LMP), and 35 frankly malignant tumours] were studied for mutation of the *nm23-H1* and the *nm23-H2* genes using single-strand conformational polymorphism (SSCP) analysis. In addition, loss of heterozygosity (LOH) at the *nm23* locus on chromosome 17q was studied by CA repeat polymorphism analysis. Mutation of the K-*ras* gene was also analysed in the same specimens. A novel mutation of the *nm23* gene was found in one case of stage III serous carcinoma without lymph model metastases. Sequencing of the subcloned mutant cDNA revealed a missense mutation from TGG to CGG at codon 133 of the *nm23-H2* gene, resulting in a change from Trp to Arg. LOH at the *nm23* locus was detected in 5 of 23 (21.7%) informative cases of ovarian carcinoma. Mutation of the K-*ras* gene was detected in 2 of 35 (5.7%) carcinomas at codons 12 and 13 respectively. There was no correlation between clinical stage or metastatic status of ovarian carcinoma and *nm23* mutation, LOH at the *nm23* locus or K-*ras* mutation. The expression levels of both the *nm23-H1* and the *nm23-H2* genes were lower in the tumour with *nm23-H2* mutation and higher in those with K-*ras* mutation. This suggests that mutation of the *nm23* genes and the K-*ras* gene affects carcinogenesis or progression of ovarian carcinoma by modulating expression of the *nm23* genes.

**Keywords:** *nm23*; nucleoside disphosphate kinase; mutation; allelic deletion; ovarian carcinoma

Ovarian carcinoma is the leading cause of death in female genital tract malignancies. Amplification and overexpression of the c-*erb*B-2 gene (Slamon *et al.*, 1989), mutational activation of the K-*ras* gene (Fukumoto *et al.*, 1989), and mutation of the p53 tumour-suppressor gene (Berchuck *et al.*, 1990; Okamoto *et al.*, 1991; Koshiyama *et al.*, 1995) are detected in ovarian cancer. The *nm23* gene was initially cloned as a metastasis-suppressor gene by differential hybridisation between low and high metastatic clones of a murine melanoma cell line (Steeg *et al.*, 1988). Two isotypes, *nm23-H1* and *nm23-H2*, have been identified in the human genome (Stahl *et al.*, 1991) and expression levels of the *nm23* genes are inversely correlated with metastatic potential in breast (Barnes *et al.*, 1991), gastric (Nakayama *et al.*, 1993) and hepatic carcinomas (Nakayama *et al.*, 1992) and malignant melanomas (Flørenes *et al.*, 1992). We previously reported mRNA levels of the *nm23* genes in ovarian tumour tissues; both *nm23-H1* and *nm23-H2* gene expressions are much higher in carcinomas than in benign tumours. Furthermore, expression of the *nm23-H1* gene is significantly lower in carcinomas possessing lymph node and or distant metastases than those without metastasis (Mandai *et al.*, 1994). However, it remains undetermined whether the changes in *nm23* expression are associated with its genetic alterations or not. Qualitative as well as quantitative alterations of the *nm23* genes such as loss of heterozygosity (LOH), amplification and point mutation have recently been reported in colon carcinoma (Cohn *et al.*, 1991; Wang *et al.*, 1993) and in neuroblastoma (Hailat *et al.*, 1991; Leone *et al.*, 1993). Accordingly, we investigated mutational changes of the transcribed *nm23-H1* and the *nm23-H2* genes in ovarian carcinoma tissues using single-strand conformational polymorphism (SSCP) analysis and sequencing of reverse transcription polymerase chain reaction (RT-PCR) products. The LOH at the *nm23* locus on chromosome 17q was examined by CA repeat polymorphism analysis. Since the product of *nm23* genes, nucleoside diphosphate (NDP) kinase, has been postulated to function as a modulator of biochemical pathways by interacting with GAP proteins (Teng *et al.*, 1991), mutation of the K-*ras* gene was investigated in the same specimens. Tumour tissues are composed of a mixture of cancer cells and normal surrounding cells and quality of RNA is not uniform among tissues. This leads to underestimation of genetic changes in tumour tissues compared with cell lines. Therefore, mutation analysis of the K-*ras* gene was also carried out to confirm the experiments in the present study by comparing the incidence with other reports. We subsequently analysed the correlation between these genetic changes and clinical stage, the metastatic status of ovarian carcinoma, and the expression level of *nm23* genes which we previously described (Mandai *et al.*, 1994).

### Materials and methods

### *Tissue samples*

Fresh surgical specimens of ovarian epithelial tumours were obtained from 41 patients who underwent oophorectomy and hysterectomy (Table I). The tissues for investigation were prepared carefully under a dissecting microscope to eliminate inappropriate components and stored at −80°C for subsequent analysis. If necessary, tumour tissues were obtained from collected 10-μm-thick frozen sections, in one of which it was determined that the tumour component exceeded 80%. They consisted of three histologically benign cystadenomas, three low malignant potential (LMP) tumours and 35 frankly invasive carcinomas. Clinical staging was performed according to the classification of the International Federation of Obstetrics and Gynecology (FIGO, 1987). In four cases of stage III, samples could be obtained both from primary tumours and peritoneal metastatic lesions. Two cell lines derived from ascites with ovarian adenocarcinomas, SK-OV-3 and NIH:OVCAR-3, were purchased from the American Type Culture Collection.

Correspondence: M Fukumoto

Received 6 January 1995; revised 11 April 1995; accepted 28 April 1995

**Table I** Patient age, clinical stage, histological type, site of metastasis, expressions, mutation and LOH of *nm23* and mutations of K-*ras* in each case of ovarian carcinoma

| *Case* | *Age* | *Stage* | *Histology* | *Metastasis* | nm23-H1 | nm23-H2 | nm23 *mutation* | *LOH at* nm23 *locus* | *K*-ras *mutation* |
|---|---|---|---|---|---|---|---|---|---|
| 1 | 42 | I | Serous (LMP) | | 0.91 | 1.77 | – | – | – |
| 2 | 55 | I | Mucinous (LMP) | | 0.51 | ND | – | ND | – |
| 3 | 61 | I | Mucinous (LMP) | | 0.40 | ND | – | ND | – |
| 4 | 51 | I | Serous | | 0.62 | 0.44 | – | NI | – |
| 5 | 56 | I | Serous | | 0.65 | 1.55 | – | – | – |
| 6 | 43 | I | Endometrioid | | 1.01 | 0.32 | – | + | – |
| 7 | 45 | I | Endometrioid | | 0.78 | 3.18 | – | – | – |
| 8 | 51 | I | Endometrioid | | 1.11 | 0.78 | – | NI | – |
| 9 | 59 | I | Endometrioid | | 1.56 | 0.62 | – | + | codon 13 |
| 10 | 60 | I | Endometrioid | | 1.12 | 1.14 | – | – | – |
| 11 | 47 | I | Clear cell | | 0.67 | 0.47 | – | – | – |
| 12 | 52 | I | Clear cell | | 0.68 | 1.19 | – | – | – |
| 13 | 40 | I | Clear cell | | 1.24 | ND | – | – | – |
| 14 | 54 | II | Serous | | 0.80 | 2.29 | – | – | – |
| 15 | 70 | II | Endometrioid | | 1.26 | 1.59 | – | – | – |
| 16 | 47 | II | Clear cell | | 0.87 | 1.70 | – | NI | – |
| 17 | 40 | III | Serous | P | 1.29 | 1.31 | – | + | – |
| 18 | 46 | III | Serous | P.L | 0.58 | 1.75 | – | – | – |
| 19 | 47 | III | Serous | P | 1.77 | 4.16 | – | – | codon 12 |
| 20 | 48 | III | Serous | P.L | 0.69 | ND | – | ND | – |
| 21 | 54 | III | Serous | P | 0.52 | 0.48 | *nm23-H2* | – | – |
| 22 | 58 | III | Serous | P.L | 0.94 | ND | – | ND | – |
| 23 | 58 | III | Serous | P.L | 0.61 | ND | – | ND | – |
| 24 | 58 | III | Serous | P | 1.26 | 1.61 | – | + | – |
| 25 | 61 | III | Serous | P | 0.50 | 0.78 | – | NI | – |
| 26 | 63 | III | Serous | P | 1.18 | 2.16 | – | NI | – |
| 27 | 63 | III | Serous | P | 0.82 | ND | – | – | – |
| 28 | 66 | III | Serous | P | 0.82 | ND | – | + | – |
| 29 | 71 | III | Serous | P | 1.72 | ND | – | NI | – |
| 30 | 46 | III | Mucinous | P.L | 0.67 | ND | – | ND | – |
| 31 | 37 | III | Endometrioid | P.L | 0.61 | ND | – | ND | – |
| 32 | 45 | III | Endometrioid | P | 0.74 | 2.38 | – | – | – |
| 33 | 49 | III | Endometrioid | P | 0.90 | 2.18 | – | – | – |
| 34 | 30 | IV | Serous | P.L.D | 0.49 | ND | – | NI | – |
| 35 | 33 | IV | Serous | P.D | 1.04 | 0.34 | – | ND | – |
| 36 | 62 | IV | Serous | P.D | 0.72 | 1.14 | – | – | – |
| 37 | 61 | IV | Serous | P.L.D | 0.33 | ND | – | ND | – |
| 38 | 19 | IV | Serous | P.L.D | 0.92 | ND | – | ND | – |
| 39 | 79 | | mucinous cystoadenoma | | 0.61 | 0.99 | – | – | – |
| 40 | 48 | | serous cystadenoma | | 0.58 | 1.22 | – | – | – |
| 41 | 27 | | serous cystadenoma | | ND | ND | – | – | – |

Expression levels of the *nm23-H1* and the *nm23-H2* genes were described previously (Mandai *et al*., 1994). ND, not determined; NI, not informative; P, peritoneal dissemination; L, lymph node metastasis; D, distant metastasis.

### *RNA preparation, cDNA synthesis, and SSCP analysis of PCR products (PCR-SSCP)*

Isolation of total RNA, preparation of complementary DNA and PCR were carried out according to the method previously described (Mandai *et al*., 1994). The initial PCR amplified 3′ and 5′ halves of cDNAs of *nm23-H1* (bases 81–356 and 319–586 respectively) and *nm23-H2* (bases 53–330 and 293–632 respectively), as described by Leone *et al*. (1993). Regions corresponding to exon 1 and exon 2 of the K-*ras* gene were amplified using a Ras gene primer set (Takara Shuzo, Kyoto, Japan). Fifteen cycles of second PCR were performed using 0.5 μl from the initial PCR in a mixture of 8 pmol per primer, 125 μmol of dNTP other than dCTP, 12.5 μmol of dCTP, 37 kBq of [$\alpha$-$^{32}$P] dCTP, and 0.1 unit of *Taq* DNA polymerase with a reaction buffer in a volume of 10 μl. Then 1 μl of the products was diluted 1:20 with a loading buffer (95% formamide, 2 mM EDTA, 0.05% bromophenol blue, 0.05% xylene cyanol) and denatured at 90°C for 5 min. Amounts of 1 μl of each aliquot were loaded onto 6% acrylamide, non-denaturing gels both with and without 10% glycerol in TBE buffer (1 M Tris, 20 mM EDTA, 1 M borate) and were electrophoresed for 4 h at 35 W with fan-cooling. Gels were subsequently dried and visualised using a BAS 2000 Bioimage Analyzer (Fujix, Tokyo, Japan).

### *Subcloning and sequencing*

The 3′ half of the *nm23-H2* gene in which mutational change was found by PCR-SSCP analysis was subcloned. The cDNA was amplified with the primers including exogenous *Eco*RI site at the 5′ ends. The product was electrophoresed in 1% agarose, purified, and subcloned into *Eco*RI site of pUC18 plasmid after *Eco*RI digestion. Clones were screened by SSCP analysis and sequenced by direct sequencing with a CircumVent thermal cycle sequencing kit (New England Biolabs, MA, USA) using the same primers for SSCP analysis labelled with [$\gamma$-$^{32}$P]ATP by T4 polynucleotide kinase.

### *Analysis of LOH*

Genomic DNA was isolated from matched pairs of tumour tissue and normal control tissue. PCR of microsatellite locus was performed according to the method of Hall *et al*. (1992). Reaction products were denatured and electrophoresed in 6% polyacrylamide gels containing 8 M urea, dried and analysed with the BAS 2000 Bioimage analyser. LOH was confirmed when the allelic ratio in tumour DNA exceeded 3:1 conpared with an allelic ratio in corresponding normal DNA normalised to 1:1.

### *Statistical analysis*

Comparison of *nm23* expressions between two groups was performed using the Mann–Whitney *U*-test. The values of *nm23-H1* and *nm23-H2* expression represent relative expression of these genes to $\beta_2$-microglubulin and $\beta$-actin expression respectively, as previously described (Mandai *et al*., 1994).

### Results

*Detection of mutation in the* nm23 *genes*

Both of the positive controls for mutant cDNA of the *nm23-H1* and *nm23-H2* genes, which were produced using primers with point mutations, showed altered migration in SSCP analysis (data not shown). This indicates that PCR–SSCP method in the present study is sensitive enough to detect one base pair mutation of the *nm23* genes.

Mutation of the *nm23-H1* or the *nm23-H2* gene was present in none of the three benign or three LMP tumours, but was detected in 1 of the 35 (2.9%) ovarian carcinomas investigated. In this tumour (case 21; Table I), altered shift of the band was detected in the 3′ half of the *nm23-H2* gene (Figure 1a). This patient had stage III, serous adenocarcinoma without lymph node metastasis. Peripheral blood leucocytes obtained from the same patient revealed the wild-type alleles for both the *nm23-H1* and the *nm23-H2* genes (data not shown). This ruled out the possibility of polymorphism in the germ line of this patient. Neither of the two ovarian cancer cell lines showed any apparent mutation of the *nm23* genes.

*Sequencing of the mutated* nm23 *genes*

The PCR product was subcloned into pUC18 plasmid, and six clones containing an insert were obtained. On SSCP analysis, three of the six clones exhibited the same mobility as the mutant allele previously detected and the remaining showed mobility of wild-type allele (Figure 1a). Sequencing analysis exhibited T to C transversion at base 469 (Trp 133 to Arg) in all three clones suspected for mutation (Figure 1b), whereas the remaining three showed no mutation.

*Analysis of LOH at* nm23 *locus*

LOH at the *nm23* locus was examined in a total of 31 ovarian tumours (three benign, one LMP and 27 carcinoma cases), in which six carcinoma patients were homozygous for *nm23* microsatellite locus. LOH at *nm23* locus was present in none of the three benign or one LMP tumours, but was detected in 5 of 21 (23.8%) informative cases of ovarian carcinoma (Table I, Figure 2). Homozygous deletion was not detected in any case examined. Of the five ovarian carcinomas with allelic deletion for the *nm23* locus, two were stage I endometrioid adenocarcinomas, and three were stage III serous carcinomas without lymph nodal metastases. In one informative case of stage III serous carcinoma (case 19), both primary tumour and peritoneal metastatic lesion were negative for LOH at the *nm23* locus. There was no significant relationship between the presence of LOH at the *nm23* locus and clinical stage or metastatic status of ovarian carcinoma. Neither of the two informative cases with lymph nodal metastases and or distant metastases exhibited LOH at the *nm23* locus.

*Mutation of the K-*ras *gene*

Mutation of the K-*ras* gene was detected in none of the three benign or three LMP ovarian tumours, but in 2 of the 35 (5.7%) frankly invasive carcinomas. One case with K-*ras* mutation was stage I endometrioid adenocarcinoma (case 9) and the other was stage III serous carcinoma without lymph nodal metastasis (case 19; Figure 3). In SSCP analysis, the ratio of radioactivity of the mutational bands to those of normal shifted bands was approximately 1:1 in case 9 and 8:1 in case 19. In case 19 the primary tumour and the peritoneal metastatic tumour were analysed and both showed the same mobility of bands (Figure 3). Direct sequencing exhibited transversion from GGC to GTC at codon 13 in case 9 and GGT to GTT at codon 12 in case 19, both of which resulted in a change from glycine to valine.

**Figure 1** Detection of mutation of the *nm23* genes. (**a**) SSCP analysis of mutation of the *nm23-H2* gene. 18, case 18 as a normal control; 21, case 21 with mutation. (1)–(4): subcloned PCR products of case 21. (**b**) Sequencing of subcloned PCR products of case 21. Clone (1) shows T to C transversion while clone (2) indicates wild-type sequence.

**Figure 2** Analysis of LOH (arrows) in paired normal (N) and tumour (T) at the *nm23* microsatellite locus. Number above each lane is patient number.

**Figure 3** SSCP analysis of K-*ras* mutation. Numbers refer to case numbers. P, primary tumour; M, metastatic tumor; Ov, OVCAR-3 cell line. The same pattern of migration is observed in primary and metastatic tumours in case 19.

*Relationship between the* nm23 *mutation, LOH at the* nm23 *locus or K-*ras *mutation and expressions of the* nm23 *genes*

The status of mutation of the *nm23* genes, LOH at *nm23* locus and K-*ras* gene mutation was analysed in relation to mRNA levels of the *nm23-H1* and the *nm23-H2* genes which were determined in our previous study (Mandai *et al*., 1994). A case with *nm23-H2* mutation showed low levels of expression for both *nm23-H1* and *nm23-H2* (Table I). With regard to LOH at *nm23* locus, mean mRNA levels in carcinomas with and without allelic deletion was not significantly different (1.19 ± 0.28 and 0.93 ± 0.36 respectively). Both of the two carcinomas with K-*ras* mutation showed higher levels of mRNA of *nm23-H1* (0.89 ± 0.30 *vs* 1.67 ± 0.15, $P = 0.022$. Mann-Whitneys *U*-test) and *nm23-H2* (not significant).

### Discussion

The present study revealed that mutation of the *nm23* genes was present in 1 of the 35 (2.9%) ovarian carcinomas investigated and in none of the three benign or three LMP tumours. There have been two reports concerning point mutation in *nm23* genes; mutation in the *nm23-H1* or *nm23-H2* genes was found in five of seven (71.4%) neuroblastomas and the tumours with mutant *nm23* genes were associated with amplification and/or overexpression of the *nm23-H1* gene and with poorer patient survival (Leone *et al*., 1993). In another report on colorectal cancer, the *nm23-H1* gene was mutated in 2 of 12 (16.7%) cases; there was no *nm23-H1* expression in one case and 64 base deletion in the *nm23-H1* transcript in the remaining case (Cohn *et al*., 1991). In our ovarian carcinoma series, mutation of the *nm23-H2* gene was detected in only one case, and there were no tumours with *nm23-H1* mutation. Therefore, the incidence of *nm23* gene mutation in ovarian carcinoma is much lower than that of neuroblastoma and colorectal carcinoma. Expressions of the *nm23* genes have been reported to show multifunctional aspects in relation to the progression and/or metastasis of human cancers; *nm23* overexpression is correlated with advanced stage disease of neuroblastoma (Hailat *et al*., 1991) and colon carcinoma (Haut *et al*., 1991). Mutation of the *nm23* genes may play an important role in the progression of these tumours, although it is not clear if mutant *nm23* has dominant negative effect for suppression of the tumour progression or metastasis. In contrast, *nm23* expression is inversely correlated with the metastatic potential in breast (Barnes *et al*., 1991), gastric (Nakayama *et al*., 1993), hepatic (Nakayama *et al*., 1992) and ovarian carcinomas (Mandai *et al*., 1994), and melanomas (Flørenes *et al*., 1992). In these tumours, reduced *nm23* expression is associated with high metastatic potential of the tumour, and point mutation of the *nm23* genes has not been reported except for one case in the present study. In our series, the remaining eight stage III cases without nodal involvement and the 11 cases with positive nodal and/or distant metastases did not exhibit mutation of the *nm23* genes. In addition, no mutation of the *nm23* genes was found in two ovarian cancer cell lines, SK-OV-3 and NIH:OVCAR-3, both of which have been established from ascitic cells of patients with advanced ovarian cancer (American Type Culture Collection, 1988). Consequently, mutation of the *nm23* genes is not a common feature of ovarian carcinoma and may not significantly contribute to its tumour progression.

The mutation of the *nm23-H2* gene found in the present study was located in codon 133, which resulted in amino acid change from Trp to Arg. This is different from that previously reported in a case of neuroblastoma (Leone *et al*., 1993), in which the mutation was located in codon 48 of the *nm23-H2* gene. An X-ray analysis for the structure of NDP kinase of a slime mould suggests that Trp-133 in human NDP kinase donates an H-bond to His-51 near the active site (Moréra *et al*., 1994). Although both Trp-133 and His-51 are conserved among *Drosophila* and vertebrates, they may not be implicated in kinase activity (Moréra *et al*., 1994). Thus, the mutation found in this study could be a random event which does not affect biological activity of NDP kinase. However, a mutation of the NDP kinase gene in *Drosophila* causes lower stability without changes of kinase activity resulting in altered interactions between NDP kinase and other proteins (Lascu *et al*., 1992), and biological relevance of the mutation in this residue remains uncertain.

It is reported that the incidence of LOH at the *nm23* locus is found in 65% of ovarian cancer and that allelic loss on chromosome 17q is higher in advanced stages of ovarian carcinoma than in the early stages (Phillips *et al*., 1993). In Japan, ovarian carcinoma is less common than in other Western countries (Tashiro *et al*., 1992) and allelic loss on chromosome 17 of Japanese ovarian cancer (Sato *et al*., 1991) is about half as frequent compared with other studies (Jacobs *et al*., 1993; Phillips *et al*., 1993). In the present study LOH at the *nm23* locus was observed in 23.8% of informative cases. This suggests that genetic changes different from those found in Western countries may also contribute to ovarian carcinogenesis in Japan. Furthermore, it may be because informative cases in the current study were composed of a relatively high proportion of early stages (stage I and II) that the incidence of *nm23* LOH was low. In colorectal carcinomas, LOH of the *nm23-H1* gene was reportedly associated with a more aggressive behaviour of the tumour (Cohn *et al*., 1991). In the present study, however, the presence of LOH at the *nm23* locus in ovarian carcinoma was not significantly correlated with its stage or metastatic status. As the number of cases investigated is limited, further study is necessary to verify the correlation between LOH at the *nm23* locus and the tumour progression, and *nm23* expression in ovarian carcinoma.

Mutation of the K-*ras* gene was found in 2 of the 35 (5.7%) ovarian carcinomas, and was localised in codon 12 and codon 13 respectively. The incidence and locations are consistent with previous reports on K-*ras* mutation in ovarian carcinomas (Fukumoto *et al*., 1989, Enomoto *et al*., 1991), indicating that cDNA preparation in the present study was adequate for analysis of expressed genes in tumour cells. The presence of K-*ras* mutation was not related with the stage or metastatic status of the tumour. In a case of stage III carcinomas, the sample from peritoneal metastasis showed the same mutation as that from the primary tumour. This suggests that the cancer cells are monoclonal as well as that the mutation of the K-*ras* gene is a relatively early event during the progression of ovarian carcinoma. Interestingly, the expression levels of both the *nm23-H1* and the *nm23-H2* genes were higher in the tumour with K-*ras* mutation compared with those without. We previously reported significantly positive correlation between *nm23-H1* expression and c-*erb*B-2 expression in ovarian carcinoma (Mandai *et al*., 1994). Since products of the *nm23* genes have been thought to play an important role as modulators of biochemical pathways either through NDP kinase activity (Teng *et al.*, 1991) or through other forms of phosphorylase activity (MacDonald *et al.*, 1993), there may be some relationship between K-*ras* mutation and increased expression of the *nm23* genes in ovarian cancer cells. The current study suggests that mutation of the *nm23* genes and the K-*ras* gene affects expression of the *nm23* gene in ovarian carcinoma and is related to its carcinogenesis or progression.

### Abbreviations

LOH, loss of heterozygosity; PCR, polymerase chain reaction; RT-PCR, reverse-transcription PCR; SSCP, single-strand conformational polymorphism; LMP, low malignant potential; NDP kinase, nucleoside disphosphate kinase.

### Acknowledgements

We thank Dr Narimichi Kimura, Department of Molecular Biology, Tokyo Metropolitan Institute of Gerontology, Tokyo, Japan, for pertinent discussion. This work was partly supported by Grants-in-Aid from the Ministry of Education, Science and Culture of Japan to MF.

### References

AMERICAN TYPE CULTURE COLLECTION. (1988). *Catalogue of Cell lines and Hybridomas*. American Type Culture Collection: Bethesda.

BARNES R, MASOOD S, BARKER E, ROSENGARD AM, COGGIN DL, CROWELL T, KING CR, PORTER-JORDAN K, WARGOTZ ES, LIOTTA LA AND STEEG PS. (1991). Low nm23 protein expression in infiltrating ductal breast carcinomas correlates with reduced patient survival. *Am. J. Pathol.*, **139**, 245-250.

BERCHUCK A, KAMEL A, WHITAKER R, KERNS B, OLT G, KINNEY R, SOPER JT, DODGE R, CLARKE-PEARSON DL, MARKS P, McKENZIE S, YIN S AND BAST JR RC. (1990). Overexpression of *HER-2 neu* is associated with poor survival in advanced epithelial ovarian cancer. *Cancer Res.*, **50**, 4087-4091.

COHN KH, WANG F, DESOTO-LAPAIX F, SOLOMON WB, PATTERSON LG, ARNOLD MR, WEIMER J, FELDMAN JG, LEVY AT, LEONE A AND STEEG PS. (1991). Association of *nm23*-H1 allelic deletions with distant metastases in colorectal carcinoma. *Lancet*, **338**, 722-724.

ENOMOTO T, WEGHORST CM, INOUE M, TANIZAWA O AND RICE JM. (1991). K-*ras* activation occurs frequently in mucinous adenocarcinomas and rarely in other common epithelial tumors of the human ovary. *Am. J. Pathol.*, **139**, 777-785.

FIGO (1987). Changes in definitions of clinical staging for carcinoma of the cervix and ovary: International Federation of Gynecology and Obstetrics. *Am. J. Obstet. Gynecol.*, **156**, 263-264.

FLØRENES VA, AAMDAL S, MYKLEBOST O, MAELANDSMO GM, BRULAND ØS AND FODSTAD Ø. (1992). Levels of *nm23* messenger RNA in metastatic malignant melanomas: inverse correlation to disease progression. *Cancer Res.*, **52**, 6088-6091.

FUKUMOTO M, ESTENSON RD, SHA L, OAKLEY GJ, TWIGGS LB, ADOCOCK LL, CARSON LF AND RONINSON IB. (1989). Association of Ki-*ras* with amplified DNA sequences, detected in human ovarian carcinomas by a modified in-gel renaturation assay. *Cancer Res.*, **49**, 1693-1697.

HAILAT N, KEIM DR, MELHEM RF, ZHU XX, ECKERSKORN C, BRODEUR GM, REYNOLDS CP, SEEGER RC, LOTTSPEICH F, STRAHLER JR AND HANASH SM. (1991). High levels of p19/nm23 protein in neuroblastoma are associated with advanced stage disease and with N-*myc* gene amplification. *J. Clin. Invest.*, **88**, 341-345.

HALL JM, FRIEDMAN L, GUENTHER C, LEE MK, WEBER JL, BLACK DM AND KING M-C. (1992). Closing in on a breast cancer gene on chromosome 17q. *Am. J. Hum. Genet.*, **50**, 1235-1242.

HAUT M, STEEG PS, WILLSON JKV AND MARKOWITZ SD. (1991). Induction of nm23 gene expression in human colonic neoplasms and equal expression in colon tumors of high and low metastatic potential. *J Natl Cancer Inst.*, **83**, 712-716.

JACOBS IJ, SMITH SA, WISEMAN RW, FUTREAL PA, HARRINGTON T, OSBORNE RJ, LEECH V, MOLYNEUX A, BERCHUCK A, PONDER BAJ AND BAST JR RC. (1993). A deletion unit on chromosome 17q in epithelial ovarian tumors distal to the familial breast/ovarian cancer locus. *Cancer Res.*, **53**, 1218-1221.

KOSHIYAMA M, KONISHI I, MANDAI M, KOMATSU T, YAMAMOTO S, NANBU K AND MORI T. (1995). Immunohistochemical analysis of p53 protein and 72 kDa heat shock protein (HSP72) expression in ovarian carcinomas. *Virchows Archiv*, **425**, 603-609.

LASCU I, CHAFFOTTE A, LIMBOURG-BOUCHON B AND VÉRON M. (1992). A Pro/Ser substitution in nucleoside disphosphate kinase of *Drosophila melanogaster* (mutation *Killer of prune*) affects stability but not catalytic efficiency of the enzyme. *J. Biol. Chem.*, **267**, 12775-12781.

LEONE A, SEEGER RC, HONG CM, HU YY, ARBOLEDA MJ, BRODEUR GM, STRAM D, SLAMON DJ AND STEEG PS. (1993). Evidence for *nm23* RNA overexpression, DNA amplification and mutation in aggressive childhood neuroblastomas. *Oncogene*, **8**, 855-865.

MACDONALD NJ, ROSA ADL, BENEDICT MA, FREIJE JMP, KRUTSCH H AND STEEG PS. (1993). A serine phosphorylation of Nm23, and not its nucleoside diphosphate kinase activity, correlates with suppression of tumor metastatic potential. *J. Biol. Chem.*, **268**, 25780-25789.

MANDAI M, KONISHI I, KOSHIYAMA M, MORI T, ARAO S, TASHIRO H, OKAMURA H, NOMURA H, HIAI H AND FUKUMOTO M. (1994). Expression of metastasis-related *nm23-H1* and *nm23-H2* genes in ovarian carcinomas: correlation with clincopathology, *EGFR*, *c-erb*B-2, and *c-erb*B-3 genes, and sex steroid receptor expression. *Cancer Res.*, **54**, 1825-1830.

MORÉRA S, LASCU I, DUMAS C, LEBRAS G, BRIOZZO P, VÉRON M AND JANIN J. (1994). Adenosine 5'-diphosphate binding and the active site of nucleoside diphosphate kinase. *Biochemistry*, **33**, 459-467.

NAKAYAMA H, YASUI W, YOKOZAKI H AND TAHARA E. (1993). Reduced expression of nm23 is associated with metastasis of human gastric carcinomas. *Jpn. J. Cancer Res.*, **84**, 184-190.

NAKAYAMA T, OHTSURU A, NAKAO K, SHIMA M, NAKATA K, WATANABE K, ISHII N, KIMURA N AND NAGATAKI S. (1992). Expression in human hepatocellular carcinoma of nucleoside disphosphate kinase, a homologue of the nm23 gene product. *J. Natl Cancer Inst.*, **84**, 1349-1354.

OKAMOTO A, SAMESHIMA Y, YOKOYAMA S, TERASHIMA Y, SUGIMURA T, TERADA M AND YOKOTA J. (1991). Frequent allelic losses and mutations of the *p53* gene in human ovarian cancer. *Cancer Res.*, **51**, 5171-5176.

PHILLIPS N, ZIEGLER M, SAHA B AND XYNOS F. (1993). Allelic loss on chromosome 17 in human ovarian cancer. *Int. J. Cancer*, **54**, 85-91.

SATO T, SAITO H, MORITA R, KOI S, LEE JH AND NAKAMURA Y. (1991). Allelotype of human ovarian cancer. *Cancer Res.*, **51**, 5118-5122.

SLAMON DJ, GODOLPHIN W, JONES LA, HOLT JA, WONG SG, KEITH DE, LEVIN WJ, STUART SG, UDOVE J, ULLRICH A AND PRESS MF. (1989). Studies of HER-2/*neu* proto-oncogene in human breast and ovarian cancer. *Science*, **244**, 707-712.

STAHL JA, LEONE A, ROSENGARD AM, PORTER L, KING CR AND STEEG PS. (1991). Identification of a second human *nm23* gene, *nm23-H2*. *Cancer Res.*, **51**, 445-449.

STEEG PS, BEVILACQUA G, KOPPER L, THORGEIRSSON UP, TALMADGE JE, LIOTTA LA AND SOBEL ME. (1988). Evidence for a novel gene associated with low tumor metastatic potential. *J. Natl Cancer Inst.*, **80**, 200-204.

TASHIRO H, MIYAZAKI K, OKAMURA H, IWAI A AND FUKUMOTO M. (1992). *c-myc* over-expression in human primary ovarian tumours: its relevance to tumour progression. *Int. J. Cancer*, **50**, 828-833.

TENG DHF, ENGELE CM AND VENKATESH TR. (1991). A product of the *prune* locus of *Drosophila* is similar to mammalian GTPase-activating protein. *Nature*, **353**, 437-440.

WANG L, PATEL U, GHOSH L, CHEN HC AND BANERJEE S. (1993). Mutation in the *nm23* gene is associated with metastasis in colorectal cancer. *Cancer Res.*, **53**, 717-720.